# Effect of Dams and Suckling Lamb Feeding Systems on the Fatty Acid Composition of Suckling Lamb Meat

**DOI:** 10.3390/ani11113142

**Published:** 2021-11-03

**Authors:** Gianni Battacone, Mondina Francesca Lunesu, Salvatore Pier Giacomo Rassu, Giuseppe Pulina, Anna Nudda

**Affiliations:** Dipartimento di Agraria, Sezione di Scienze Zootecniche, University of Sassari, Viale Italia 39, 07100 Sassari, Italy; battacon@uniss.it (G.B.); pgrassu@uniss.it (S.P.G.R.); gpulina@uniss.it (G.P.); anudda@uniss.it (A.N.)

**Keywords:** suckling lamb, meat fatty acid, grass, maternal diet

## Abstract

**Simple Summary:**

Suckling lamb meat is one of the most relevant products of the Mediterranean traditional dairy sheep industry. Being lamb fed exclusively with maternal milk, meat quality is mainly affected by the mother’s milk composition and hence by the mother feeding during gestation and lactation. This paper summarizes the state of the art about the effect of the dam and suckling lamb feeding systems on lamb meat quality, with special attention to the lipid fraction considered beneficial to human health.

**Abstract:**

The effects of the dams and suckling lamb feeding systems on the fatty acid (FA) profile of lamb meat are reviewed in this article. The suckling lamb can be considered a functional monogastric, and therefore, its meat FA composition is strongly influenced by the FA composition of maternal milk. The major source of variation for ewe milk FA composition is represented by pasture amount and type. In the traditional sheep breeding system of the Mediterranean area, the main lambing period occurs in late autumn–early winter, and ewes are able to exploit the seasonal availability of the natural pastures at their best. Therefore, lambs start suckling when maternal milk concentrations of vaccenic, rumenic, and n-3 long-chain polyunsaturated FA in maternal milk are the highest. When maternal diet is mainly based on hay and concentrates, the use of vegetable oils can be considered a good strategy to improve the meat FA profile of suckling lambs.

## 1. Introduction

Sheep productions (milk, meat, and wool) are among the principal agricultural activities of the Mediterranean Area, where they play a prominent socio-economic role because of their strong link with the territory [1]. Despite its ancient origin, the current sheep industry is characterized by a high level of multifunctionality, integrating food security and safety, animal welfare, environmental sustainability, ecosystem services, and biodiversity preservation. World meat production, including sheep meat, is expected to double by 2050 [2], due to the growing population and incomes. Thus, special attention should be paid to meat quality.

Suckling lamb meat is a typical product of the Mediterranean countries where dairy sheep farming is widespread, with milk as the main and lamb meat as the secondary products [3,4]. Suckling lambs are fed only maternal milk, and they are slaughtered at 4–6 weeks of age with a body weight of 9–11 kg [3,5,6,7]. Even if the traditional suckling is the most common, artificial suckling with milk replacer or partial suckling systems are sometimes used to increase the amount of milk available for cheese processing and to improve lamb survival [8,9].

The suckling lamb can be considered a functional monogastric due to the underdevelopment of rumen. Thus, meat quality strongly depends on maternal milk composition [3,10,11]. For this reason, research on dairy sheep breeds has focused on milk nutritional and technological properties in order to improve firstly the quality of dairy products [1] but also of suckling lamb meat. The fat and fatty acids (FA) profiles of maternal milk strongly affect meat FA profile with consequences on its nutritional and sensory characteristics. Sheep milk is rich in potentially healthy FA, especially rumenic (RA; C18:2c9,t11; also named c9,t11-conjugated linoleic acid or c9,t11-CLA), vaccenic (VA; C18:1t11), linolenic (ALA; C18:3n3) [12], and branched-chain fatty acids (BCFA) [13].

Currently, there is a growing number of health-aware consumers that require meat with less fat, lower levels of saturated fatty acids (SFA), and higher content of FA of nutritional interest. It is widely assessed that the intramuscular FA profile of suckling lambs reflects that of their mother’s milk. Thus, the diet composition during pregnancy [14] and/or lactation [3,7,15,16,17] can affect meat characteristics.

For this reason, several studies on the modification of fat content and FA composition of lamb meat have been carried out. The role of this review is to provide advanced updates about relationships between the FA profile of suckling lamb meat and feeding systems of dams and suckling lambs aimed at improving the ewe-milk-fed suckling lamb meat.

## 2. The Quality of Sucking Lamb Fat

Suckling lamb meat represents an important source of lipids and provides some FA that are involved in many biological activities connected to the health status [18], especially essential FA [19] and specific long-chain polyunsaturated fatty acids (LC-PUFA), which are crucial for normal fetal development and neonatal growth [20,21]. In addition, lamb is the first meat recommended by Italian pediatricians in the weaning diet of children because it is presumed to have lower allergenicity, compared with other red meats [22,23].

Among the different types of meat, suckling lamb meat contains a low amount of intramuscular fat (Table 1; around 2–3%), which are composed of 41–46% of SFA, 42–43% of monounsaturated fatty acids (MUFA) and 15–11% of PUFA [3,24]. On the basis of this composition, it can be classified as extra-lean meat as defined by FDA [25], with less than 5 g of fat and 2 g of SFA in 100 g of meat, respectively. The predominant FA in suckling lamb meat are oleic (OLA; C18:1c9), followed by palmitic (PA; C16:0) and stearic (SA; C18:0) acids, in line with the FA profile of commercial lamb meat from the typical production systems of Italy [26,27,28], Spain, Germany, United Kingdom, and Uruguay [29].

The high content of OLA in suckling lamb meat is of nutritional interest because of its positive role in the prevention of cardiovascular, autoimmune, and inflammation diseases [31,32]. The content of SFA in suckling lamb meat, composed mainly by PA and SA in similar proportion, is lower than that of heavier and adult sheep (Table 1). Recently, the role of all SFA in human health has been reconsidered because it has been reported that all types of FA, including SFA, are not related to cardiovascular disease mortality, and saturated fat is inversely associated with stroke [33]. In addition, it has been assessed that PA, which accounts for 20–30% of total human body FA and that was for a long time associated with negative health effects [18], has several essential and positive physiological properties [34]. SA, which accounted for about 6.5–13% of total FA in the meat of suckling lamb [3,26], can be considered neutral for cholesterol metabolism probably because it is rapidly converted into OLA [35,36].

Regarding the unsaturated FA (UFA) profile, suckling lamb meat has a sufficient amount of both n3 and n6 FA and its n6/n3 ratio is close to the optimal value of 5:1 (Table 1), which is markedly lower than that typical of Western diet (15:1 to 20:1) [37]. Moreover, the level of LC-PUFA is higher than in fatter or heavier lambs (Table 1) or in adult sheep. In fact, PUFA of both the omega3 and omega6 families are preferentially incorporated in membrane phospholipids rather than in triglycerides [38], and the proportion of phospholipids on total lipids decreases with the age and fatness of the animals [39]. For the same reason, the proportion of this LC-PUFA increases in cooked meat due to the loss during cooking, mainly of triglycerides fraction that contains comparatively more SFA than UFA [3]. The meat of suckling lamb contains a higher amount of ALA, arachidonic (ARA; C20:4n6), eicosapentaenoic (EPA; C20:5n3; 13-fold higher), docosapentaenoic (DPA; C22:5n3), docosahexaenoic (DHA; C22:6n3; 4-fold higher) acids and, in general, of PUFA of the omega3 family (PUFAn3) than the meat of adult sheep [28]. Arachidonic acid, as a constituent of phospholipids membrane, is important for the function of all cells [40] and, together with DHA, favors the development and function of the nervous and visual system [20]. However, ARA metabolites could contribute both to cardiovascular and neurodegenerative diseases [41]. The ARA and DHA derive directly from the FA of the diet or through elongation and desaturation of their dietary precursors ALA and linoleic acid (LA; C18:2n6), respectively [42]. Although most scientific researchers have focused on PUFA, the role of other FA should not be neglected. Noteworthy FA in suckling lamb meat are the odd-chain FA (OCFA) and BCFA. These FA are considered biomarkers of rumen microbial fermentation and their amount in the meat of suckling lambs, which are still functional monogastric, is likely related to their concentration in the maternal milk [13]. The odd- and branched-chain fatty acids (OBCFA), not considered for a long time because of their low content in the total fat, have recently received increasing attention in human nutrition due to their potential health properties. The BCFA tested in animal models showed anti-inflammatory properties [43,44], and antitumoral activity in human breast cancer cells [45,46]. The main OCFA, C17:0 and C15:0 showed protective effects against cardiovascular disease [47,48,49,50,51] and contrast occurrence of type 2 diabetes [52,53,54,55]. A recent study demonstrated that C15:0 has a direct role in attenuating multiple comorbidities in humans as inflammation, anemia, and dyslipidemia [56]. Lamb meat, together with dairy foods, represents the main source of both OCFA and BCFA in the human diet [13].

Meat represents also one of the main sources of VA and c9,t11-CLA in the human diet. The content of c9,t11-CLA in suckling lamb meat is in line with those reported in other studies on suckling [26] and weaned lambs [38]. Clinical and animal studies have suggested anti-inflammatory and immunomodulatory antiatherogenic activities of c9,t11-CLA [57,58,59], as well as lowering cholesterol [60,61,62] and hyperinsulinemia prevention [63,64] effects. The anticarcinogenic effect of c9,t11-CLA has been widely observed in several studies on cell cancer lines and laboratory animals [58], but these results need to be confirmed in humans.

Changes in the UFA level in meat lead to the production of different amounts and types of volatile compounds (VOCs), which can affect the sensory properties of suckling lamb meat. The oxidation of PUFA, present in high proportion in suckling lamb meat, might produce aldehydes, as pentanal and hexanal from LA and ALA, heptanal and octanal from OLA and LA, and nonanal from OLA [65]. These compounds are usually related to the overall positive acceptability of flavor, because they have low odor detection thresholds [66].

## 3. Suckling Lamb Feeding System and Meat Fatty Acid Profile

### 3.1. Suckling Lambs from Ewes Grazing on Pasture

Meat and milk derived from animals reared in pasture-based farming systems are highly appreciated by the consumers due to the perception of greater welfare of the grazing animals and to the superior quality of animal-derived products [67]. This is true especially for the suckling lamb of the Mediterranean area, where consumer responses highlighted that meat of lambs nursed by mothers fed on pasture was preferred to meat from lambs of indoor-fed mothers [67,68].

**Figure 1:**
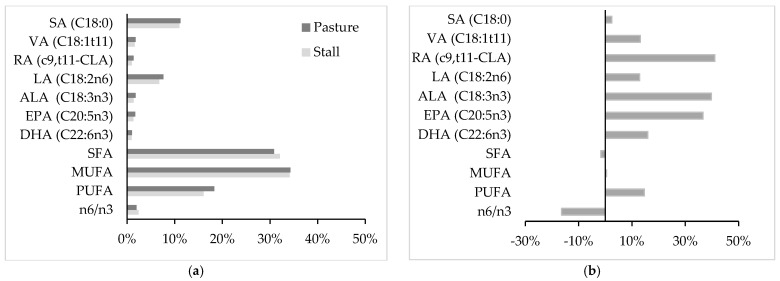
Effect of pasture- or stall-fed mothers on intramuscular fatty acid profile of suckling lamb meat. −Data are reported as % of total fatty acids (**a**) and as the proportional difference (%) between the pasture group and the control group (**b**). SA = stearic acid (C18:0), VA = vaccenic acid (C18:1t11), RA = rumenic acid (c9,t11-CLA), LA = linoleic acid (C18:2n6), ALA = α-linolenic acid (C18:3n3), EPA = eicosapentaenoic acid (C20:5n3), DHA = docosahexaenoic acid (C22:6n3), SFA = saturated fatty acids, MUFA = monounsaturated fatty acids, PUFA = polyunsaturated fatty acids. References: [11,67,70,71,72,73].

From a nutritional point of view, suckling lamb meat from pasture-fed mothers exhibits an FA profile more favorable for human health than suckling lamb meat from stall-fed mothers (hay and concentrate) (Figure 1). In particular, meat of lambs of pasture-fed mothers shows a larger content of PUFA, especially of the n3 family including ALA but also LC-PUFAn3, with a marked reduction in n6/n3 ratio. In addition, an increase in VA and RA has been also detected in lambs from pasture-fed mothers. These differences are partly related to maternal milk FA composition (Table 2), and they depend on the transfer from grass to milk [69], then to the meat of suckling animals, of the compounds beneficial for human health.

Plants are the primary source of essential PUFAn3, especially ALA, which is partly bio-hydrogenated into VA in the rumen and then secreted into milk and partially converted into c9,t11-CLA in the mammary tissue by the action on VA by stearoyl–CoA desaturase [75]. The essential ALA present in milk is partly incorporated into tissues of suckling lambs as is. On the other hand, milk has a very limited content of LC-PUFA due to the high level of rumen biohydrogenation [76], to the low activity of elongation and desaturation in the mammary gland of FA with 18 and 20 carbon atoms, and also because these LC-PUFA are not transported with the plasma lipid fractions, which are the main mammary sources of FA uptake [77]. However, muscle tissue—even if at a limited extent—has the capacity to further metabolize ALA to EPA and DHA [78] by ∆6 desaturases and ∆5 desaturases, respectively, determining the observed favorable increase in LC-PUFA (Figure 1b).

However, the pasture effects on milk and, consequently, on meat c9,t11-CLA, and PUFAn3 contents depend on the seasonal variation of forage quality and the botanical composition of herbage [79,80]. In terms of seasonal variations, the best FA composition of grass can be reached during the vegetative phase, whereas during the reproductive phase the concentration of ALA falls dramatically [80,81]. The quantity and quality of pasture availability for ewes could be the underlying reason for variation in the FA profile of suckling lamb meat reared in different seasons [7]. This last study reported an interesting variation in OCFA and BCFA, which were higher in meat from lambs reared in spring than winter season (4.72 vs. 3.95 g/100 g of fat, respectively). The intake of small amounts of herbage by the suckling animals that follow the mother on pasture might occur early during spring than winter season, causing an earlier development of the forestomach fermentation system. Surprisingly, no variation in PUFAn3 has been observed in the meat of suckling lambs reared in winter or spring.

In view of these considerations, the traditional Mediterranean breeding system, where lambing occurs in late autumn–early winter allows ewes to exploit the seasonal availability of the natural and cultivated pastures. Therefore, lambs are mainly nursed when maternal milk’s FA contents of nutritional interest such as VA, RA, PUFAn3, LC-PUFA, and OBCFA are at the highest level.

### 3.2. Suckling Lambs Fed Maternal Milk or Milk Replacers

Artificial rearing based on the use of milk replacers is used to increase the amount of commercial milk sold to cheese-making companies [9,82].

The composition of lamb meat reflects that of maternal milk (Table 3), which, compared with commercial milk replacers, is richer in c9,t11-CLA, VA, ALA, EPA, DHA [8,24,83,84], and also in OBCFA, which are from three- to tenfold higher [24].

From a nutritional and healthy point of view, suckling lamb meat, compared with artificially reared ones (Table 4), presents a more favorable nutritional FA profile since it contains more c9,t11-CLA (+79%), VA (+760%), ALA (+256%), EPA (+188%), and DHA (+117%). On the other hand, the use of artificial rearing increases the total PUFA [8,10] because most of the fat components of artificial milk are derived from vegetable oils (coconut or palm oils), which are characterized by a lower level of saturation, compared with animal fats. In addition, artificial rearing also increases the tocopherol content that is commonly added as an ingredient in the milk replacer [85].

Although the ratio of PUFA/SFA is higher in the artificially than naturally reared lambs [8,10,83], maternal milk presents an acceptable range of n6/n3 ratio than that of milk replacer, which generally reaches values above the recommended maximum of 4 [10,86].

In view of these considerations, the traditional rearing system remains the best strategy to enhance the milk FA profile and the lamb meat composition from a nutritional point of view through the manipulation of the mother’s diet.

Nevertheless, in our previous study, few differences were found in the meat of lambs fed with both maternal milk (only at night) and concentrate (during the day, compared with lambs fed with only maternal milk [9]—the lack of differences was not a surprise, as both groups received milk from pasture-fed mothers.

### 3.3. Suckling Lambs from Ewes Fed Diets Containing Vegetable or Marine Oils

The dietary supplementation of ewes during pregnancy or at lambing with vegetable or marine oils could positively affect ewe and lamb fatty acid status, neonatal behavior and growth performance of lambs, and colostrum production [87,88]. However, the use of oils, particularly when the mother’s diet is based mainly on hay and concentrates or whenever the availability and the quality of pasture is low, could be also an effective strategy to improve the meat FA profile of suckling lambs. However, the information found in the scientific literature is quite inconsistent, and studies deal mainly with linseed supplementation.

**Figure 2:**
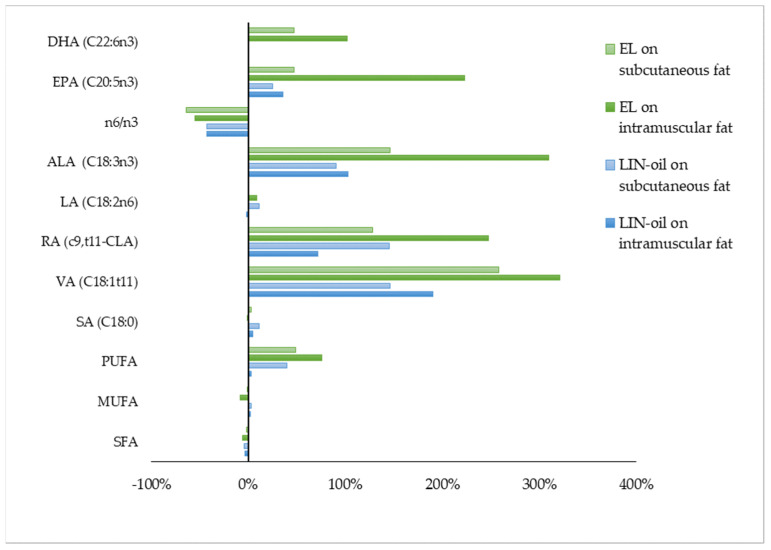
Effect of linseed oil (LIN-oil) or extruded linseed (EL) on intramuscular and subcutaneous fatty acid profile of suckling lamb meat. Data are reported as the proportional difference (%) between the treatment group and the control group. DHA = docosahexaenoic acid (C22:6n3), EPA = eicosapentaenoic acid (C20:5n3), ALA = α-linolenic acid (C18:3n3), LA = linoleic acid (C18:2n6), RA = rumenic acid (c9,t11-CLA), VA = vaccenic acid (C18:1t11), SA = stearic acid (C18:0), PUFA = polyunsaturated fatty acids, MUFA = monounsaturated fatty acids, SFA = saturated fatty acids. References: [89,90].

Linseed is the most widely used lipid supplement to test the effect of maternal diet on FA profile of intramuscular [11,17,89,90] and subcutaneous fat of suckling lamb because linseed is rich in ALA [12]. Linseed inclusion, both as oil or in extruded form, strongly ameliorates the FA profile, especially at the intramuscular fat level (Figure 2; Table 5).

In addition to linseed supplementation, the inclusion of olive oil and soybean oil in the diet of ewes is able to increase the content of OLA and LA in the intramuscular fat of suckling lambs [89].

Other studies evinced that the inclusion of calcium in ewes’ diet is effective to increase the content of SFA, especially PA, in the intramuscular fat of suckling lambs [89,91].

Finally, the inclusion of marine oils, a source of LC-PUFA, in the diet of ewes noticeably improved the content of EPA and DHA, but also that of c9,t11-CLA, and VA, in the intramuscular fat of suckling lambs [6]. However, the inclusion of marine oils and the excessive increase in PUFA in meat can adversely affect flavor and meat oxidative stability. These potential detrimental effects on flavor and shelf life should be considered when diets of ewes are formulated to improve nutritional features in lamb meat.

## 4. Conclusions

Suckling lambs’ meat presents a FA composition that reflects that of the suckled milk. Suckling lamb meat, compared with that from artificially reared lambs, presents a more favorable nutritional FA profile since it contains more c9,t11-CLA, ALA, and LC-PUFA of the n3 family. From a nutritional point of view, suckling lamb meat from pasture-fed mothers has interesting and distinguishing qualitative traits. When maternal diet is mainly based on hay and concentrate, the supplementation of vegetable oils, especially linseed or marine oils to the mothers, can be a strategy to ameliorate the meat FA profile of suckling lambs.

## Figures and Tables

**Table 1:** Fatty acid composition of intramuscular lipid (% of total fatty acids) in suckling lambs from the extensive system, heavy lambs reared in the intensive system, and mature ewes.

Item ^1^	Suckling Lambs ^2^	Heavy Lamb ^3^	Mature Ewes ^4^
Fat content, %	1.93	2.86	3.27
C14:0 (Myristic acid, MA)	3.72	4.41	1.85
C14:1c9	0.15	0.13	0.10
isoC14:0	0.04	0.05	0.02
isoC15:0	0.12	0.10	0.12
anteisoC15:0	0.18	0.17	0.13
C15:0	0.45	0.52	0.42
C15:1c10	0.00	n.d. ^5^	0.15
isoC16:0	2.60	0.16	0.07
C16:0 (Palmitic acid, PA)	16.53	20.57	24.47
C16:1c7	0.48	0.47	0.29
C16:1c9	1.23	1.44	1.16
isoC17:0	0.51	0.40	0.46
anteisoC17:0	0.40	0.48	0.60
C17:0	0.76	1.22	1.22
C17:1c9	0.43	0.47	0.46
isoC18:0	n.d.	0.12	0.15
C18:0 (Stearic acid, SA)	12.16	16.82	18.22
C18:1t9 (Elaidic acid, EA)	0.22	0.37	0.18
C18:1t10	0.36	3.75	0.32
C18:1t11 (Vaccenic acid, VA)	1.86	1.48	2.01
C18:1t12	0.00	0.84	0.00
C18:1c9 (Oleic acid, OLA)	29.20	30.94	37.47
C18:1c11	1.42	1.10	0.97
C18:1c12	0.44	0.47	0.21
C18:1c13	0.10	0.14	0.11
C18:1c14	0.00	0.08	
C18:1c15	0.14	0.09	0.18
C18:2n6 (Linoleic acid, LA)	8.84	4.98	4.34
C18:3n3 (Linolenic acid, ALA)	1.97	1.03	0.92
c9,t11-CLA (Rumenic acid, RA)	1.16	0.62	0.84
C20:2n6	0.08	0.04	0.04
C20:4n6 (Arachidonic acid, ARA)	3.63	1.07	0.04
C20:3n3	0.05	0.01	0.97
C20:5n3 (Eicosapentaenoic acid, EPA)	1.16	0.23	0.07
C22:0	0.04	0.03	0.00
C22:4n6	0.23	0.08	0.05
C22:5n3 (Docosapentaenoic acid, DPA)	1.45	0.34	0.18
C22:6n3 (Docosahexaenoic acid, DHA)	0.86	0.13	0.00
SFA	38.21	43.76	46.84
MUFA	37.62	43.69	53.04
PUFAn3	5.58	1.78	1.17
PUFAn6	13.44	7.78	4.77
OCFA	1.22	1.76	1.66
BCFA	3.86	1.47	1.49
n6/n3	2.60	4.08	4.14

^1^ SFA = saturated fatty acids; MUFA = monounsaturated fatty acids; PUFAn3 = polyunsaturated fatty acids omega3; PUFAn6 = polyunsaturated fatty acids omega6; OCFA = odd-chain fatty acids; BCFA = branched-chain fatty acids; n6/n3 = omega6/omega3 ratio. ^2^ Source: [3,7,26]. ^3^ Source: [30]. ^4^ Source: [28]. ^5^ n.d. = not determined.

**Table 2:** Milk fatty acid profile of pasture- and stall-fed mothers.

Breed	Treatment	Fatty Acid Composition of Ewe Milk (% on Total Fatty Acids) ^1^															
		PA (C16:0)	SA (C18:0)	VA (C18:1t11)	RA (c9,t11-CLA)	LA (C18:2n6)	ALA (C18:3n3)	EPA (C20:5n3)	DHA (C22:6n3)	SFA	MUFA	PUFA	PUFA/SFA	n6	n3	n6/n3	References
Churra Tensina	Hay Pre-partum	21.04	13.30	-	1.36	-	1.44	0.11	0.07	56.15	31.34	5.80	0.10	2.54	1.61	1.62	[73]
	Pasture prepartum	20.37	14.07	-	1.56	-	1.54	0.106	0.072	56.39	30.20	6.09 ↑	0.11	2.48	1.71	1.47	
	Hay post-partum	21.17 ↑	12.96	-	1.29	-	1.4	0.104	0.074	56.37	31.61	5.70	0.10	2.53	1.58	1.64 ↑	
	Pasture postpartum	20.24	14.40 ↑	-	1.63 ↑	-	1.57 ↑	0.11	0.068	56.17	29.93	6.19 ↑	0.11 ↑	2.48	1.74 ↑	1.46	
Churra Tensina	Hay fed mothers	-	-	-	1.29	-	-	-	-	56.37	31.61	5.70	0.10	2.53	1.58	1.64 ↑	[72]
	Pasture fed mothers	-	-	-	1.63 ↑	-	-	-	-	56.17	29.93	6.19 ↑	0.11 ↑	2.48	1.74 ↑	1.46	
Merino	Stall fed mothers	22.14	9.59	1.23	1.00	3.28	2.60	-	-	66.88	24.33	8.80	0.13	-	-	-	[71]
	Pasture fed mothers	18.38	11.48	1.70 ↑	1.33	3.11	4.46 ↑	-	-	65.26	23.89	10.85 ↑	0.17 ↑	-	-	-	
Massese	Stall (reared indoor fed concentrate and hay)	25.15	8.60	-	-	2.35	0.87	0.13	0.09	73.10	22.07	3.95	-	2.86	1.09	-	[74]
	Semi free-range (reared indoors during the night, fed concentrate, hay, and herbage)	23.57	12.41	-	-	2.73	1.38	0.18	0.09	70.94	23.19	4.78	-	3.13	1.65	-	
	Pasture (reared outdoor and fed pasture and hay)	23.71	15.85 ↑	-	-	2.62	2.18 ↑	0.23	0.23 ↑	63.18 ↓	29.33 ↑	6.09 ↑	-	3.43	2.66 ↑	-	

↑ = increased, (*p* < 0.05); ↓ = decreased, (*p* < 0.05). ^1^ PA = palmitic acid (C16:0); SA = stearic acid (C18:0); VA = vaccenic acid (C18:1t11); RA = rumenic acid (c9,t11-CLA); LA = linoleic acid (C18:2n6); ALA = α-linolenic acid (C18:3n3); EPA = eicosapentaenoic acid (C20:5n3); DHA = docosahexaenoic acid (C22:6n3); SFA = saturated fatty acids; MUFA = monounsaturated fatty acids; PUFA = polyunsaturated fatty acids; n6/n3 = omega6/omega3 ratio.

**Table 3:** Fatty acid composition of maternal milk and milk replacer.

Type of Milk	Fatty Acid Composition of Suckled Milk (% on Total Fatty Acids) ^1^										References
	SA (C18:0)	RA (c9,t11-CLA)	LA (C18:2n6)	ALA (C18:3n3)	EPA (C20:5n3)	DHA (C22:6n3)	SFA	MUFA	PUFA	n6/n3	
Maternal milk	12.43	-	1.55	1.40	n.d.	0.15	66.37	20.07	6.19	1.64	[10]
Milk replacer	13.56	-	4.71	0.49	0.91	0.13	60.23	30.11	9.66	4.39	
Maternal milk	10.74	1.23	1.66	2.81	-	-	72.38	21.01	5.69	0.59	[8]
Milk replacer	10.14	0.50	8.30	1.07	-	-	66.15	23.27	10.12	7.95	
Maternal milk	10.10	0.99	2.85	0.97	-	-	69.77	24.36	5.76	3.37	[24]
Milk replacer	11.44	0.38	5.60	0.25	-	-	60.04	33.66	7.20	24.21	
Maternal milk	10.43	4.93	2.90	0.94	0.05	0.04	61.49	31.60	1.22	2.71	Our data
Milk replacer	4.17	0.04	8.67	1.18	n.d.	n.d.	52.67	35.56	1.26	7.66	

^1^ SA = stearic acid (C18:0); RA = rumenic acid (c9,t11-CLA); LA = linoleic acid (C18:2n6); ALA = α-linolenic acid (C18:3n3); EPA = eicosapentaenoic acid (C20:5n3); DHA = docosahexaenoic acid (C22:6n3); SFA = saturated fatty acids; MUFA = monounsaturated fatty acids; PUFA = polyunsaturated fatty acids; n6/n3 = omega6/omega3 ratio; n.d. = not detected.

**Table 4:** Intramuscular fatty acid profile of meat from suckling lambs of maternal or artificial milk.

Breed	Type of Rearing System	Fatty Acid Composition of Intramuscular Suckling Lamb Meat (% on Total Fatty Acids) ^1^											References
		SA (C18:0)	VA (C18:1t11)	RA (c9,t11-CLA)	LA (C18:2n6)	ALA (C18:3n3)	EPA (C20:5n3)	DHA (C22:6n3)	SFA	MUFA	PUFA	n6/n3	
Comisana	Artificial	14.00	-	-	8.48	0.42	0.18	0.17	43.2	43.2	13.6	9.5	[10]
	Traditional	12.83	-	-	4.05	1.37	0.62	0.45	46.6	41.8	11.6	2.0	
Sarda	Artificial	11.33	-	-	10.51	0.53	0.21	0.28	43.9	38.7	17.3	9.9	[86]
	Traditional	11.37	-	-	7.63	0.69	0.40	0.35	50.3	34.5	15.2	6.0	
Barbaresca	Artificial	11.64	-	0.47	18.47	0.37	0.80	0.53	34.1	33.3	32.6	9.7	[8]
	Traditional	12.02	-	1.13	10.97	1.95	1.65	1.25	37.7	34.9	27.4	2.6	
Churra	Artificial	8.37	-	0.67	8.01	0.12	0.30	-	34.0	49.9	16.3	16.3	[24]
	Traditional	12.53	-	0.51	8.44	1.30	1.44	-	40.5	39.9	19.7	5.2	
Slovak	Artificial	10.14	0.11	0.15	9.07	0.25	0.06	0.08	44.7	42.5	12.7	3.5	[83]
	Traditional	12.68	0.96	0.67	4.81	0.72	0.35	0.25	45.6	43.3	11.1	1.4	
Sarda	Artificial	8.47	0.00	0.08	15.56	0.82	0.45	0.52	35.3	40.8	23.9	8.0	Our data
	Traditional	13.07	2.34	1.63	5.08	1.26	0.66	0.59	44.6	44.8	10.6	2.1	
Average	Artificial	10.66	0.06	0.34	11.68	0.42	0.33	0.26	39.2	41.4	19.4	9.5	
	Traditional	12.42	1.65	0.98	6.83	1.22	0.85	0.48	44.2	39.8	15.9	3.2	

^1^ SA = stearic acid (C18:0); VA = vaccenic acid (C18:1t11); RA = rumenic acid (c9,t11-CLA); LA = linoleic acid (C18:2n6); ALA = α-linolenic acid (C18:3n3); EPA = eicosapentaenoic acid (C20:5n3); DHA = docosahexaenoic acid (C22:6n3); SFA = saturated fatty acids; MUFA = monounsaturated fatty acids; PUFA = polyunsaturated fatty acids; n6/n3 = omega6/omega3 ratio.

**Table 5:** Effect of maternal diet based on vegetable oils on intramuscular fatty acid profile of suckling lamb meat. Data are reported as the proportional difference (%) between the supplemented group and the non-supplemented control group.

Breed	Dietary Treatment ^1^	Dose ^2^	Intramuscular Fatty Acids of Suckling Lamb Meat ^3^										References
			VA	RA	LA	ALA	EPA	DHA	SFA	MUFA	PUFA	n6/n3	
Churra	Olive oil	63 g/d	57%	3%	−8%	−16%	−13%	57%	−5%	11%	−7%	7%	[89]
	Soya oil	63 g/d	176%	97%	7%	−19%	−32%	176%	−6%	7%	0.5%	26%	
	Linseed oil	63 g/d	190%	71%	−2%	103%	35%	190%	−4%	2%	3%	−43%	
Churra	Extruded linseed	189 g/d	321%	247%	9%	309%	223%	102%	−6%	−9%	76%	−56%	[90]
Churra	LO (with 3% linseed oil)	3% linseed oil	361%	224%	−6%	56%	−10%	−39%	−6%	10%	−5%	−16%	[17]
	LO-Syn E	LO plus 400 mg/kg TMR of synthetic vitamin E	363%	208%	21%	95%	90%	24%	−14%	8%	29%	−23%	
	LO-Nat E	LO plus 400 g/kg TMR of natural vitamin E)	446%	226%	−4%	69%	3%	−37%	−7%	9%	−2%	−21%	
Comisana	Extruded linseed	190 g/d	-	47%	-	-	-	-	−7%	0.13%	17%	−31%	[11]

^1^ LO = linseed oil; Syn E = synthetic vitamin E; Nat E = natural vitamin E. ^2^ TMR = Total mixed ration. ^3^ VA = vaccenic acid (C18:1t11); RA = rumenic acid (c9,t11-CLA); LA = linoleic acid (C18:2n6); ALA = α-linolenic acid (C18:3n3); EPA = eicosapentaenoic acid (C20:5n3); DHA = docosahexaenoic acid (C22:6n3); SFA = saturated fatty acids; MUFA = monounsaturated fatty acids; PUFA = polyunsaturated fatty acids; n6/n3 = omega6/omega3 ratio.

## Data Availability

The data presented in this study are available on request from the corresponding author.
